# Immune Responses to HBsAg Conjugated to Protein D of Non-Typeable *Haemophilus influenzae* in Mice

**DOI:** 10.1371/journal.pone.0117736

**Published:** 2015-02-17

**Authors:** Qiudong Su, Yao Yi, Feng Qiu, Xuexin Lu, Junying Ding, Zhiyuan Jia, Ruiguang Tian, Yan Gao, Shengli Bi

**Affiliations:** National Institute for Viral Disease Control and Prevention, China Center for Disease Control and Prevention, Beijing, China; University of Melbourne, AUSTRALIA

## Abstract

**Background:**

Hepatitis B vaccine that contains an aluminum hydroxide adjuvant induces apoptotic death of Hepa 1–6 cells. Difficult-to-degrade chemical additives in vaccines effectively enhance vaccine immunogenicity, but also affect the host tissue. Identification of bio-molecules that are readily degraded and compatible *in vivo* as an adjuvant is important for vaccine research. The hapten–carrier effect suggests that stimulation of helper T (Th) cells by carrier adjuvants is feasible. Protein D (PD) of non-typeable *Haemophilus influenzae* covalently conjugated to some polysaccharide vaccines has been confirmed to convert T-cell independent (TI) antigens into T-cell dependent (TD) antigens, and elicit strong T-cell responses ultimately. Herein, we would substitube PD for aluminum hydroxide adjuvant in Hepatitis B vaccine.

**Methods and results:**

Truncated PD (amino acids 20–364) was expressed in *Escherichia coli* and purified by (NH_4_)_2_SO_4_ precipitation and DEAE chromatography. After evaluation of antigenicity by western blotting, PD was covalently conjugated to yeast-derived recombinant HBsAg by cross-linking with glutaraldehyde. Intramuscular immunization with the conjugate induced higher level of HBsAg-specific antibody than did HBsAg alone (*p* < 0.05), and was comparable to commercial Hepatitis B vaccine. During the surveillance period (days 35–105), anti-HBs titers were hold high. Moreover, the conjugated vaccine enhanced Th1 immune responses, while Th2 responses were also activated and induced an antibody response, as determined by IFN-γ ELISPOT and IgG1/IgG2a ratio assays.

**Conclusions:**

Recombinant truncated PD covalently conjugated to HBsAg antigen enhanced the immunogenicity of the antigen in mice simultaneously by humoral and cellular immune response, which would facilitate therapeutic hepatitis B vaccines.

## Introduction

Commercial Hepatitis B vaccine with an aluminum hydroxide as adjuvant has been widespread used over the past three decades because of safety and effectiveness in preventing HBV infection. However, the inclusion of chemical additives—aluminum hydroxide—brings in some side-effects. Hamza *et al*. found that exposure of Hepa1–6 cells to a low dose of Hepatitis B vaccine resulted in loss of mitochondrial integrity, apoptosis induction, and cell death [1]. Medical additives are being increasingly investigated (particularly if they enhance potency), particularly those that are not readily degraded *in vivo*. Moreover, the prophylactic Hepatitis B vaccine does not induce an effective immune response against HBV in chronic hepatitis B (CHB) patients with impaired T-cell immune responses to HBV antigens [2, 3] in whose body the regeneration of T cell function seems to be crucial. As a result, the T–B interaction specific to HBsAg, which is necessary for antibody production, could not occur. Thus, vaccines that mainly stimulate Th2-cell immune responses could not work.

HBV antigens (HBsAg, *etc*.) can be bound by specific antibodies (anti-HBs) *in vitro*, but are not immunogenic alone. In terms of hapten–carrier effect, HBV antigens may actually be a hapten in CHB patients. However, if HBsAg (hapten) is coupled to a protein (carrier) that can stimulate helper T (Th) cells, the conjugates could induce antibody responses against HBsAg and also possibly break tolerance to HBV antigens, for which both hapten-specific B cells and protein (carrier)-specific helper T cells are required [4]. HBsAg-specific B cells capture the conjugate by recognizing the “α” determinant; this is followed by endocytosis and presentation of peptides derived from the carrier to carrier-specific helper T lymphocytes. In this situation, the T–B interaction recognizes different epitopes of the same molecule. Thus, HBsAg activation of the T–B cell interaction requires more potent carriers that can stimulate Th cell activation.

PD is an antigenically conserved, surface-localized outer membrane protein of all *Haemophilus influenzae* species, including non-typeable (NT) *H*. *influenzae*, and is the first NT *H*. *influenzae* antigen to induce protective responses in humans [5]. PD is a promising vaccine candidate against experimental NT *H*. *influenzae* infection, and has been used as an antigenically active carrier protein. Experiments in rats revealed that vaccination with PD induced high serum IgG and IgA levels, as well as significant bactericidal activity against homologous and heterologous strains [6]. Moreover, PD has been used as a carrier protein to allow the capsular polysaccharide (T-cell independent (TI) antigens) to function as a T-cell dependent (TD) antigen. Covalently coupled to PD, the *H*. *influenzae* serotype b capsular polysaccharide induced a vigorous TD immune response and immunological memory in infants [7]. Thus, PD in conjugated vaccines can stimulate Th cell activation. In a randomized controlled trial involving infants, a 10-valent pneumococcal NT *H*. *influenzae* PD-conjugate vaccines (PHiD-CV) was shown to induce an immune response to all included pneumococcal serotypes and PD [8]. In a clinical trial involving children [9], PD was used as a carrier protein in an 11-valent pneumococcal conjugate investigational vaccine, which achieved significant protection against acute otitis media caused by pneumococci or NT *H*. *influenzae*.

In this study, we conjugated recombinant PD to HBsAg to stimulate Th cells and hopefully could enhance the immunogenicity of the vaccine by inducing T–B cell interactions. The immune response stimulated by the conjugated vaccine was evaluated in mice.

## Materials and Methods

### Construction of plasmids expressing truncated PD

The gene encoding amino acids (aa) 20–364 of PD was amplified from chromosomal DNA of NT *H*. *influenzae* by polymerase chain reaction (PCR) using *Pfu* DNA polymerase (Promega, WI, USA). The specific primers synthesized by Sangon Biotech (Shanghai, China) were 5’- GGAATTCCATATGAGCAGCCATTCATC-3’ (forward) and 5’- CCGCTCGAGTTATTTTATTCCTTT-3’ (reverse). After an initial denaturation step at 95°C for 8 min, all reactions were subjected to 35 cycles of denaturation at 95°C for 55 s, annealing at 58°C for 55 s, and extension at 72°C for 1 min, with a final extension at 72°C for 10 min. After double-enzyme digestion with *Nde*I and *Xho*I, gel-extracted 1050-bp PCR products were ligated into pET-43.1a vector with the identical cohesive terminal. The ligation reaction mixture was transformed into *Escherichia coli* strain BL21 (DE3). Ampicillin-resistant colonies were isolated and identified by restriction endonuclease analysis of the plasmid, small-scale expression, and sequencing.

### Expression and purification of truncated PD

*E*. *coli* BL21 (DE3) freshly transformed with the expression plasmid were inoculated into LB medium (10 g/l tryptone, 5 g/l yeast extract, 10 g/l NaCl) containing 50 μg/ml ampicillin at 37°C. When the OD_600_ reached 0.9, expression was induced by adding isopropylthio-D-galactoside (IPTG) to a final concentration of 1 mM, and incubated for an additional 3 h at 37°C. After harvesting by centrifugation (3,000 *g*, 10 min, 4°C), the bacterial pellet was resuspended in lysate buffer (10 mM Tris–HCl, 0.5% Triton X-100, pH 8.0) and subjected to sonication. The total bacterial proteins, supernatant and inclusion bodies were separated by centrifugation (17,400 *g*, 10 min, 4°C) and resolved in a 13.5% SDS-PAGE gel to assess the expression and form of PD. PD was purified by DEAE chromatography, onto which the soluble fraction of (NH_4_)_2_SO_4_ precipitation after dialysis was loaded. Protein concentration was determined using the Bicinchoninic Acid Protein Assay Kit (Sigma, MO, USA). Purified PD (10 μl) was then resolved in a 13.5% SDS-PAGE gel to assess the homogeneity of the purified protein.

### Western blotting

Purified PD was subjected to 13.5% SDS-PAGE and then transferred onto nitrocellulose membranes (Amersham, Sweden) at 15 V for 30 min using a Trans-blot SD semi-dry transfer cell (Bio-Rad, CA, USA). After blocking nonspecific antibody sites with 5% (m/v) Difco skim milk (BD, MD, USA) in TBST (20 mM Tris-HCl, 500 mM NaCl, pH 7.5), the nitrocellulose membrane was reacted with *H*. *influenzae* antiserum type b (1:20; BD, MD, USA), followed by anti-mouse IgG horseradish peroxidase (HRP)-conjugated secondary antibody (1:5000; Sigma, MO, USA). After washing with TBST three times and TBS finally, substrate solution containing 3, 3′-diaminobenzidine tetrahydrochloride (DAB; Sigma, MO, USA) was added and the reaction was quenched with distilled water.

### Yeast-derived recombinant HBsAg and commercial hepatitis B vaccine

The yeast-derived HBsAg (*adr*, 220 mg/ml), Commercial Hepatitis B vaccine and aluminum hydroxide adjuvant were generous gifts from Sinovac Research & Development Co., Ltd. (Beijing, China).

### HBsAg-PD conjugation

Yeast-derived HBsAg and PD were dialyzed into the conjugated buffer (1 mM DTT, 100 mM NaCl, 2 μM EDTA, 0.05% (v/v) NP-40, 10% (v/v) glycerol, 25 mM HEPES, pH 8.0). First, 25% (v/v) glutaraldehyde (Sigma, MO, USA) was added dropwise to the PD in the conjugated buffer to a final concentration of 1.25% and hold at room temperature (RT) with gentle stirring overnight. The reaction solution was loaded onto a Sephadex G-2 column and eluted with physiological saline. Finally the brown effluent was collected. HBsAg was added dropwise to the brown effluent with gentle stirring at RT for 3 h. Finally, the crosslinking reaction was quenched with Tris-glycine buffer (1 M Tris-HCl, 1 M glycine, pH 7.5). Conjugates were concentrated by (NH_4_)_2_SO_4_ precipitation and dissolved in PBS. After dialysis, the conjugate was stored at -4°C.

### Evaluation of the coupling effect by double-antibody sandwich ELISA

The 96-well ELISA plates (Nunc, Roskilde, Denmark) were coated with 100 μl of anti-HBs monoclonal antibody (2.5 μg/ml) in 50 mM sodium bicarbonate buffer (pH 9.6) overnight at 4°C. After blocking with 5% (m/v) Difco skim milk in PBST (3.2 mM Na_2_HPO_4_, 0.5 mM KH_2_PO_4_, 1.3 mM KCl, 135 mM NaCl, 0.05% Tween 20, pH 7.4) for 1 h at 37°C, 100 μl of serial diluted conjugated vaccine were added to each well and incubated for 30 min at 37°C. The wells were then washed five times, followed by addition of *H*. *influenzae* antiserum type b (1:100) and incubated for 30 min at 37°C. The wells were washed five times again, followed by addition of anti-mouse IgG horseradish peroxidase (HRP)-conjugated secondary antibody (1:5000) and incubated for 30 min at 37°C. After another five washes, 100 μl of peroxidase tetramethylbenzidine substrate (TMB) (Pharmingen, CA, USA) were added to each well. The reaction was stopped with 2 M H_2_SO_4_. The absorbance at 450 nm (OD_450_) was measured using a spectrophotometer (Thermofisher, Vantaa, Finland). All measurements were performed in triplicate. HBsAg alone was used as the negative control, and the cut-off value was calculated as 2.1-times the mean of the negative control value (if the value < 0.05, then it was reported as 0.05). The maximum dilution that yielded a positive result was regarded as the coupling potency of the conjugate vaccine.

### Mice and immunization

Specific-pathogen-free, female Balb/c mice aged 6–8 weeks were purchased from Vital River Laboratories (Beijing, China). All mice were maintained under specific-pathogen-free conditions at the Laboratory Animal Center, China CDC, and all studies were approved by the Animal Care and Welfare Committee at the National Institute for Viral Disease Control and Prevention, China Center for Disease Control and Prevention (NO. 2013035). To evaluate the conjugated vaccine, six mice per group were immunized on days 0, 14 and 21 by intramuscular (i.m.) administration of 100 μl of conjugated vaccine, commercial vaccine or HBsAg alone, each containing 5 μg HBsAg. Mice were bled by the retro-orbital route once every 7 days from day 14 to 105. After holding for 2 h at RT, serum was collected by centrifugation (3000 *g*, 20 min, RT) and stored at -20°C. Mice were sacrificed at day 35 and splenocytes were isolated for enzyme-linked immunospot (ELISPOT) assay.

### ELISPOT assay

IFN-γ and IL-4-secreting antigen-specific cells were quantified using an ELISPOT kit (BD, CA, USA), as described previously [10]. Splenocytes (5×10^5^) were added to multiscreen 96-well filtration plates pre-coated with the anti-mouse IFN-γ or IL-4 capture antibody with 10 μg/ml of corresponding antigens or 5 μg/ml of Con A (positive control) in triplicate wells. Notably, the splenocytes of the mice that received the conjugated vaccine were stimulated with PD and HBsAg respectively. The spots were counted using an automated ELISPOT reader. A response was considered positive if the number of spot-forming cells (SFC) per 5×10^5^ splenocytes was greater than 82 (IL-4) or 43 (IFN-γ). SFC values are presented as means ± standard deviation.

### Detection of anti-HBs antibodies by ELISA

The mouse hepatitis B virus surface antigen (HBsAg) ELISA kit was used according to the manufacturer’s instructions (Cusabio, China). Briefly, 100 μl of serially diluted serum were added to each well, followed by 50 μl of HRP-conjugated solution. The wells were incubated for 30 min at 37°C, and washed five times with wash buffer. Substrate A (50 μl) and B (50 μl) were added to each well, and then incubated in the dark for 15 min at 37°C. Finally, 50 μl of Stop solution were added to each well and the OD_450_ was measured using a spectrophotometer (Thermo fisher, Vantaa, Finland). The cut-off value was calculated based on 2.1-times the mean of the negative control value (if the value < 0.05, then it was reported as 0.05). We regarded the maximum dilution of positive results as the antibody titer and used log_2_ (IgG titers) to analyze the kinetics of serum anti-HBs antibody.

### IgG1/IgG2a ratio

IgG1 and IgG2a are regarded as indicators of Th2 and Th1 cell responses, respectively. We performed IgG subclass ELISAs as follows. After capturing the anti-HBs antibody on Cusabio ELISA pre-coated plates of Mouse Hepatitis B virus Surface Antibody (HBsAb) ELISA Kit (Cusabio, China), the isotype-specific reagents (1:1000) of Mouse Monoclonal Antibody Isotyping Kit (Sigma, MO, USA) were added, followed by peroxidase-conjugated rabbit anti-goat IgG affinity-isolated antibody (Sigma, MO, USA). We used log_2_ (IgG1 titers)/log_2_ (IgG2a titers) to evaluate the Th1/Th2-cell immune responses.

### Flow cytometry

Relative proportions of CD4^+^ T and CD8^+^ T cells in mouse splenocytes were analyzed by flow cytometry. Briefly, splenocytes (1×10^6^/well) derived from the conjugated vaccine and commercial vaccine groups were cultured for 5 h and co-cultured with 10 μg/ml of conjugated vaccine or commercial vaccine. Next, 10 μl of FITC-conjugated rat anti-mouse CD8 antibody and PE-conjugated rat anti-mouse CD4 antibody (eBioscience, San Diego, USA) were added and incubated in a 100 μl volume for 20 min at RT. The splenocytes were washed twice with PBS and resuspended in 500 μl PBS for flow cytometry (FACSCalibur, BD, USA).

### Statistical analysis

Statistical analysis was performed using the ANOVA test. A *P* value of less than 0.05 was considered to indicate significance.

## Results

### Construction of plasmids expressing truncated PD

**Fig 1:**
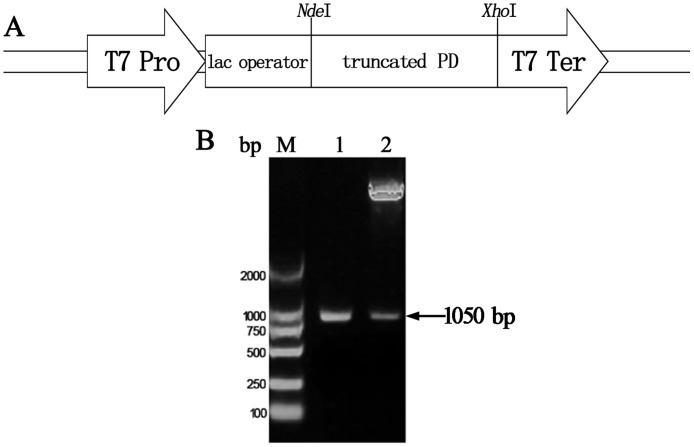
Schematic diagram of the truncated PD gene in the pET43.1a expression vector, PCR amplification of the truncated PD gene, and restriction endonuclease analysis of the expression plasmid. (A) Schematic diagram of the expression plasmid. (B) Agarose gel electrophoresis of the truncated PD gene amplified by PCR and restriction endonuclease analysis of the plasmid. Lane M, DNA marker DL2000; lane 1, truncated PD gene amplified by PCR; lane 2, expression plasmid digested with *Nde*I/*Xho*I. (T7 pro, T7 promoter; T7 ter, T7 terminator).

**Fig 2:**
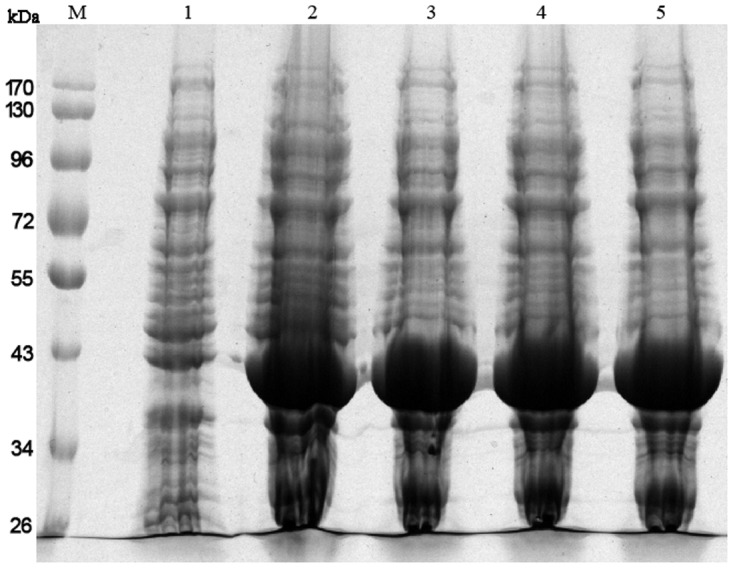
SDS-PAGE analysis of the small-scale expression of truncated PD. Lane M, pre-stained protein molecular weight marker; lane 1, negative control; lane 2–5, expression of different single-colonies.

Using specific primers, the gene fragment encoding truncated PD was amplified from chromosomal DNA of NT *H*. *influenzae* by PCR (Fig. 1B). The expression plasmid was identified by restriction endonuclease analysis (Fig. 1B) and sequencing. Moreover, small-scale expression of PD in *E*. *coli* showed that the molecular weight of the foreign protein was approximately 40.13 kDa, as expected, confirming successful construction of plasmids encoding the truncated from of PD (Fig. 2) and the percentage of the foreign protein in the total bacterial protein was approximately 42.4%.

### Expression and purification of truncated PD

**Fig 3:**
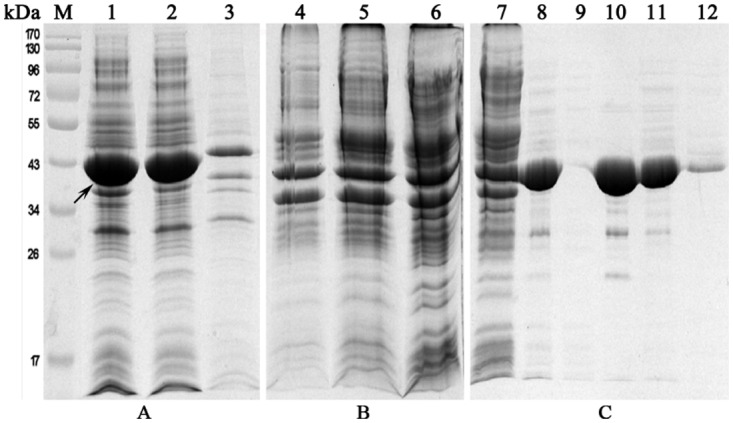
SDS-PAGE analysis of the large-scale PD expression and purification steps. (A) Distribution of PD in induced bacteria after large-scale expression. Lane M, pre-stained protein molecular weight marker; lane 1, total bacterial proteins after sonication; lane 2, soluble fraction of the sonicate; lane 3, insoluble fraction of the sonicate. (B) (NH_4_)_2_SO_4_ precipitation. Lanes 4–6, precipitated proteins at 33, 40 and 45% (NH_4_)_2_SO_4_ saturation, respectively. (C) Fine purification of PD by DEAE chromatography. Lane 7, precipitated proteins at 45% (NH_4_)_2_SO_4_ saturation; lane 8, soluble fraction at 45% (NH_4_)_2_SO_4_ saturation following dialysis; lane 9, flow-through; lane 10, elutes washed with 100 mM NaCl in buffer A; lane 11, elutes washed with 200 mM NaCl in Buffer A; lane 12, elutes washed with 400 mM NaCl in Buffer A. Arrows indicate PD.

After deleting 19 aa at the N-terminus of protein D and optimizing the expression conditions, truncated PD was expressed in soluble form in *E*. *coli*, as confirmed by the fact that PD was detected primarily in the supernatant of the sonicate (Fig. 3A), suggesting the compatibility with the host. Using (NH_4_)_2_SO_4_ precipitation, we found that the proteins precipitated at 33%, 40% and 45% (NH_4_)_2_SO_4_ saturation were contaminating bacterial proteins, leaving PD in the soluble fraction (Fig. 3B and 3C). Owing to chemical potential energy penetration, the volume of the soluble fraction dialyzed in Buffer A (10 mM Tris–HCl, pH 8.0) increased by 40.1%, while PD was dominant (Fig. 3C). After DEAE chromatography, PD almost bound with the media (Fig. 3C) was eluted in 100 mM NaCl in Buffer A (93.5% Purity) and 200 mM NaCl in Buffer A (82.6% Purity) (Fig. 3C). Roughly calculated, the PD yield was about 91 mg per liter LB medium.

### Western blotting of truncated PD

**Fig 4:**
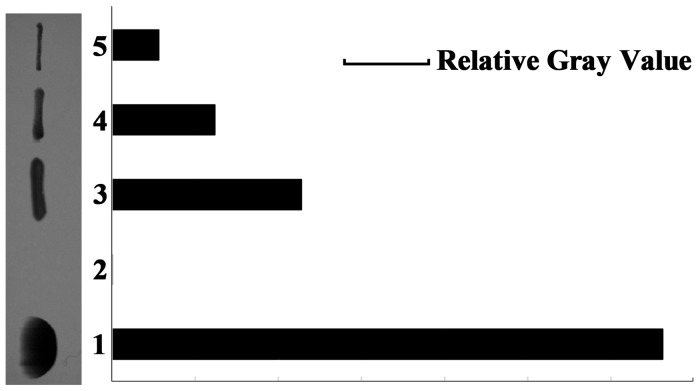
Western blotting analysis of PD and schematic diagram of the dose-response relationship. Lane 1, 10 μl of sonicate from induced bacteria; lane 2, 10 μl of sonicate from non-induced bacteria; lanes 3–5, 1:10, 1:20, and 1:40 dilutions of eluate in 100 mM NaCl in Buffer A.

Western blotting showed a specific signal band at ~40.13 kDa, as expected, with *H*. *influenza*e antiserum type b as the primary antibody (Fig. 4). Based on analysis of band intensities, we identified a dose-response relationship between PD and *H*. *influenza*e antiserum type b (Fig. 4), suggesting that the actual antigenic valence of truncated PD expressed in *E*.*coli* was considerable.

### The antigenic valence of HBsAg-PD conjugation

We employed two antibodies that respectively recognize HBsAg and PD in the conjugate to establish a double-antibody sandwich ELISA and evaluate the effect of Glutaraldehyde-mediated conjugation. After capturing the conjugate using an anti-HBs antibody, anti-PD serum (included in *H*. *influenzae* antiserum type b) and anti-mouse secondary antibody-HRP were added to detect the conjugate. Based on serial dilutions of the conjugate, we determined that the antigenic valence was 1: 1600.

### ELISPOT

**Fig 5:**
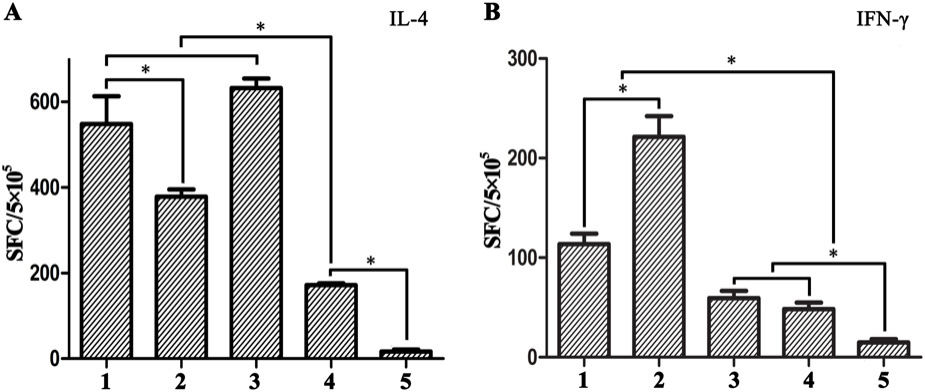
In vitro IL-4 and IFN-γ production by splenocytes of vaccinated mice stimulated by HBsAg or PD. (A) IL-4 responses determined by ELISPOT assay. 1–2, HBsAg and PD-specific IL-4 responses of splenocytes from the conjugated vaccine group, respectively; 3–4, HBsAg-specific IL-4 responses of splenocytes isolated respectively from the commercial vaccine and HBsAg alone groups; 5, PBS group. (B) IFN-γ responses determined by ELISPOT assay. 1–2, HBsAg and PD-specific IFN-γ responses of splenocytes from the conjugated vaccine group, respectively; 3–4, HBsAg-specific IFN-γ responses of splenocytes isolated respectively from the commercial vaccine and HBsAg alone groups; 5, PBS group. The symbol “*” represents statistical significant difference between the groups in the segment ends.

**IL-4**. In *ex vivo* stimulation assays, splenocytes from all experimental groups secreted IL-4 following stimulation with HBsAg or PD (Fig. 5A). Compared to immunization with HBsAg alone, immunization with the conjugated or commercial vaccine activated a greater number of HBsAg-specific IL-4-secreting lymphocytes. Splenocytes from the conjugated vaccine group also secreted IL-4 following stimulation with PD, although greater stimulation was achieved using HBsAg (*p* < 0.05). Moreover, there was no significant difference between the conjugated vaccine and commercial vaccine groups (*p* > 0.05); splenocytes from both groups exhibited greater IL-4 secretion than the HBsAg group (*p* <0.05).

**IFN-γ**. We performed IFN-γ ELISPOT assays to evaluate the frequency of HBsAg/PD-specific T cells in the spleens of vaccinated mice. IFN-γ-secreting splenocytes from the commercial vaccine and HBsAg alone groups following stimulation with HBsAg were not significantly different compared with the negative control (*p* > 0.05). However, splenocytes from the conjugated vaccine group stimulated with PD secreted a considerable amount of IFN-γ, as did splenocytes stimulated with HBsAg (Fig. 5).

### Kinetics of serum anti-HBs antibody titers

**Fig 6:**
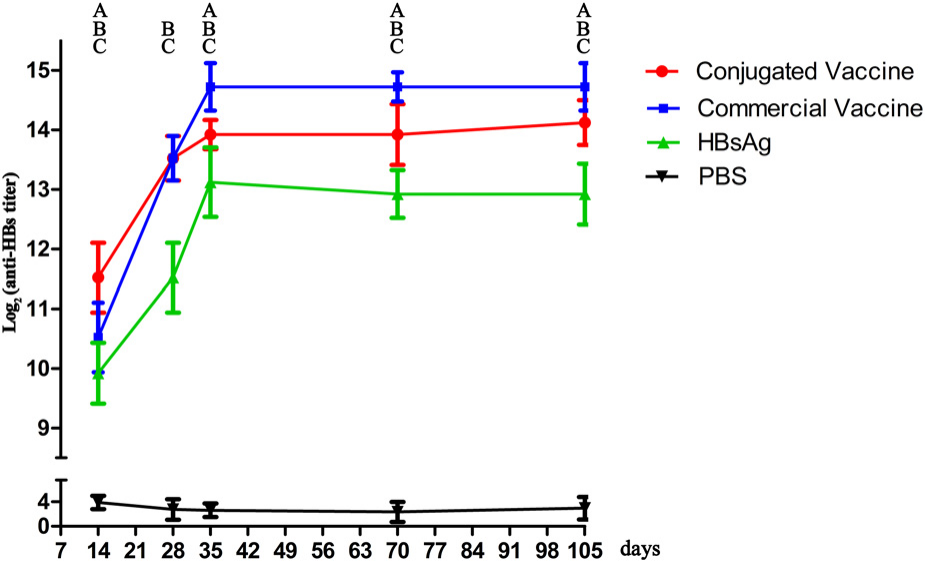
Kinetics of serum anti-HBs antibody. The symbol “A” represents statistical significant difference between conjugated vaccine and commercial vaccine groups; The symbol “B” represents statistical significant difference between conjugated vaccine and HBsAg-alone groups; The symbol “C” represents statistical significant difference between commercial vaccine and HBsAg-alone groups.

All vaccine components stimulated production of various titers of anti-HBs antibody (Fig. 6). Although the antibody titer induced by the conjugated vaccine was lower than that of the commercial vaccine, both were higher than HBsAg alone (*p* < 0.05). Notably, the antibody titer of the conjugated vaccine group was higher than that of the other two groups at day 14, which suggested that the immune response with PD as an adjuvant may be more effective in the first-needle immunization. From day 35 to 105, the anti-HBs antibody titers in all groups were unchanged.

### IgG1/IgG2a ratio

**Fig 7:**
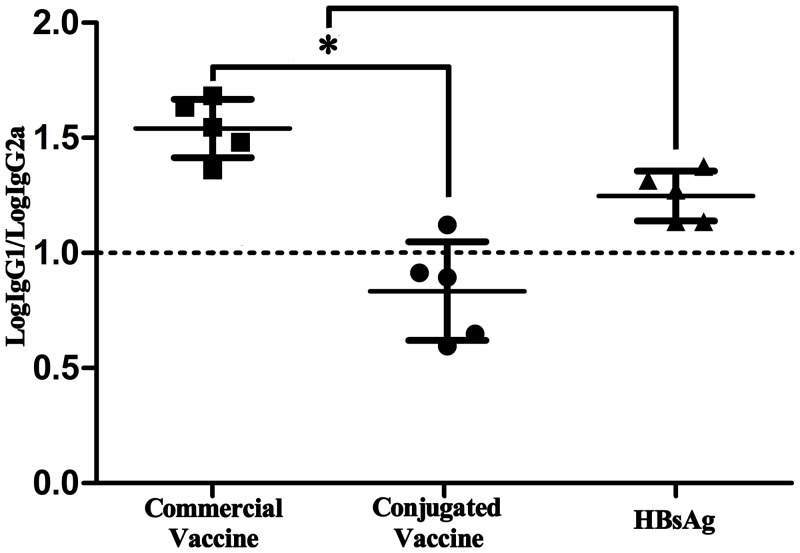
Serum IgG1/IgG2a ratios in the experimental groups. The symbol “*” represents statistical significant difference between the groups in the segment ends.

Vaccination with commercial vaccine and HBsAg alone yielded a mean IgG1 to IgG2a ratio of >1, suggesting a predominant Th2 immune response (Fig. 7A). The conjugated vaccine yielded a mean IgG1 to IgG2a ratio of ~ 0.8, demonstrating that a strong Th1 cell immune response was also stimulated (Fig. 7B). Moreover, the IgG1/IgG2a ratio differed significantly between the conjugated vaccine and commercial vaccine groups (*p* < 0.05). These results suggested that specific B cell activation differed significantly between the conjugated and commercial vaccines.

### Flow cytometry detection of CD4/CD8 T cells in splenocytes

**Fig 8:**
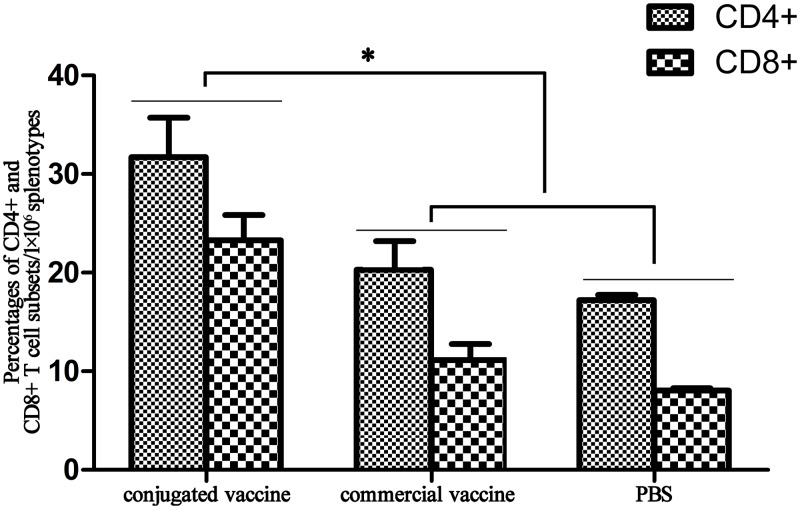
Proportions of CD4+ and CD8+ T cell subsets in the splenotype-immunized conjugated vaccine, commercial vaccine and PBS groups.

Both CD8+ CTL and CD4+ Th cells are essential for therapeutic hepatitis B vaccines. Our results (Fig. 8) showed that the proportions of CD4+ and CD8+ T cell subsets were significantly higher in mice immunized with conjugated vaccine than those immunized with commercial vaccine (*p* < 0.05), which suggested that the conjugated vaccine could activate both CD4+ and CD8+ T cell proliferation more effectively. Furthermore, the CD4+/CD8+ T cell ratio in mice immunized with the conjugated vaccine was 1.42, compared with 2.14 in the control group, indicating that the conjugated vaccine induced greater activation of CD8+ T cells than CD4+ T cells.

## Discussion

A vaccine containing HBsAg filaments can induce immune responses to all three-envelope antigen components [11]. Yeast-derived recombinant HBsAg is immunogenic, safe and cost-effective for the prevention of hepatitis B virus infection in combination with an aluminum hydroxide adjuvant [12, 13]. However (although a potent B cell stimulator), aluminum is ineffective in inducing a Th1 response and has adjuvant effects on synthetic peptides, as well as other disadvantages [14–17]. T cell induction is facilitated by pre-S antigens, which are important for the production of anti-HBs [18]. T-cell activation requires presentation of HBsAg peptides, which must be processed by antigen-presenting cells (APCs) prior to display on the surface of APCs in association with HLA antigens [19], for which strong T-cell epitopes are required. Moreover, the survival of memory B cells is dependent on specific T-cell help. Replacing the aluminum hydroxide adjuvant with biomolecules with strong T-cell epitopes is practical to establish and maintain immune memory. Furthermore, more immunogenic vaccines are required for defined groups such as non-responders or travelers and health-care workers, who require protection after only a priming dose of vaccine. The fact that conjugate adjuvants and HBsAg together elicit strong cellular and humoral immunity may allow this technique to be applied to these groups. After the initial immunization, the antibody titer of the conjugated vaccine group increased rapidly (Fig. 6), which suggested effective activation of specific B cells by the conjugated vaccine. This also demonstrated the superiority of single-dose immunization with the conjugated vaccine.

PD has unique properties, such as surface localization, a high degree of conservation, wide distribution, and pathogenicity. Because of these properties, PD has shown promising preclinical results [7–9]. PD was selected to function as an active carrier protein for HBsAg. We deleted 19 amino acids at the N-termini of PD, which contains a consensus bacterial signal peptide with a stretch of hydrophilic and basic amino acids, followed by a hydrophobic region of 13 residues with a glycine in the hydrophobic core to ensure soluble expression in *E*. *coli*. The putative signal peptide ends with the consensus sequence for lipoproteins, Leu-Ala-Gly-Cys, which is associated with the acylation reaction and proteolytic processes [20]. Apart from the hydrophobic putative signal sequence, PD is hydrophilic and contains no typical membrane-spanning region [21]. Thus, we retained only aa 20–364 to maximize the yield of soluble PD with a native conformation. To produce naked PD lacking tags to meet the requirements for a vaccine antigen, we introduced the termination codon “TAA” before the *Xho*I restriction site (Fig. 1A). The purification procedure ensured production of high-purity, soluble PD without any tags.

Although some modifications were made to ensure soluble expression, western blotting showed that the immunodominant epitopes of protein D were still remained (Fig. 4). By covalently conjugating PD to HBsAg with glutaraldehyde, we successfully constructed a hapten–carrier conjugate, which effectively activated the T-B cell interaction as demonstrated by the more rapid and specific antibody production compared to HBsAg alone. Moreover, ELISPOT assays and the IgG1/IgG2a ratio demonstrated that the Th1-cell immune response was stimulated by the conjugated vaccine, while Th2 cells were also activated to promote antibody responses. HBsAg-specific B cells bound the conjugate through the “α” determinant, followed by endocytosis, and presented T-cell epitopes derived from PD to PD-specific helper T lymphocytes. That is, the T-B interaction recognized different epitopes of the conjugated vaccine candidate. By allowing for the T-B interaction, HBsAg-specific B cells were activated and produced anti-HBs antibodies, which was very likely to break tolerance to HBV antigen in CHB patients owing to the immune response induced by PD conexisted with HBsAg in a molecule. The results of flow cytometric analysis showed that the conjugated vaccine activated CD8+ T cells to a greater degree than CD4+ T cells, while the conjugated vaccine could activate both CD4+ and CD8+ T cell subsets, which was crucial to eliminate the infected cells. The evaluation of conjugated vaccines in CHB animal models would require further study, although the preliminary results in common animal models were promising. Hopefully, the conjugated vaccine could restore impaired T cell responses in by breaking T-cell tolerance to HBV antigens.

In summary, we established a practical and large-scale procedure for production of recombinant truncated PD as the raw material for antigen-conjugated vaccine candidates. Moreover, we demonstrated the adjuvant effect of the truncated PD in HBsAg-conjugated PD vaccine which could effectively stimulated an immune response to HBsAg. These results demonstrated the possibility of using PD as an alternative adjuvant for antigens with weak T-cell epitopes, including the hepatitis B vaccine.
